# Genetic Anomalies in Pediatric Orthopedics: A Case Study of a New Rare Sporadic Mutation of Osteogenesis Imperfecta

**DOI:** 10.7759/cureus.64909

**Published:** 2024-07-19

**Authors:** Rahaf E Farah, Rou’a E Farah, Mays K Najjar, Raneen W Atatrah, Ghadeer I Eideh, Shadi A Abuisneina

**Affiliations:** 1 College of Medicine and Health Science, Palestine Polytechnic University, Hebron, PSE; 2 Pediatric Orthopedic Department, Alia Hospital, Hebron, PSE

**Keywords:** case report, col1a2, substitution, sporadic mutation, osteogenesis imperfecta

## Abstract

Osteogenesis imperfecta (OI) arises from a collagen type 1 defect due to several gene mutations, particularly COL1A1 and COL1A2. Its inheritance pattern is typically autosomal dominant, which is more common, or autosomal recessive, although sporadic cases also occur. Prenatal ultrasound can detect severe types, but genetic testing is necessary for confirmation, often at birth or in early childhood. We present a rare case of sporadic OI type III involving a three-year-old boy. Prenatal ultrasound initially revealed limb deformities and skeletal dysplasia, with subsequent confirmation at birth through bone deformities and multiple fractures. Exome sequencing confirmed the diagnosis at 15 months, revealing a new, rare variant in the COL1A2 gene. Pamidronate treatment began at seven months.

## Introduction

Osteogenesis imperfecta (OI) manifests as weak bones and fractures, with potential impacts on other tissues, such as the skin, tendons, and the sclera of the eyes [1]. It follows various inheritance patterns and typically presents in infancy, occurring in approximately one in 20,000 cases [2]. The condition results from either a de novo variant or an inherited pathogenic variant, with mosaicism detected in up to 16% of families [3,4].

Parents can undergo molecular genetic testing for at-risk pregnancies if an affected family member carries a known causative variant in COL1A1 or COL1A2 [5]. Prenatal ultrasound serves as a useful tool for detecting severe and lethal forms of OI before 20 weeks of gestation [5]. This article introduces a case featuring a rare, likely pathogenic de novo heterozygous mutation in the COL1A2 gene.

## Case presentation

**Figure 1:**
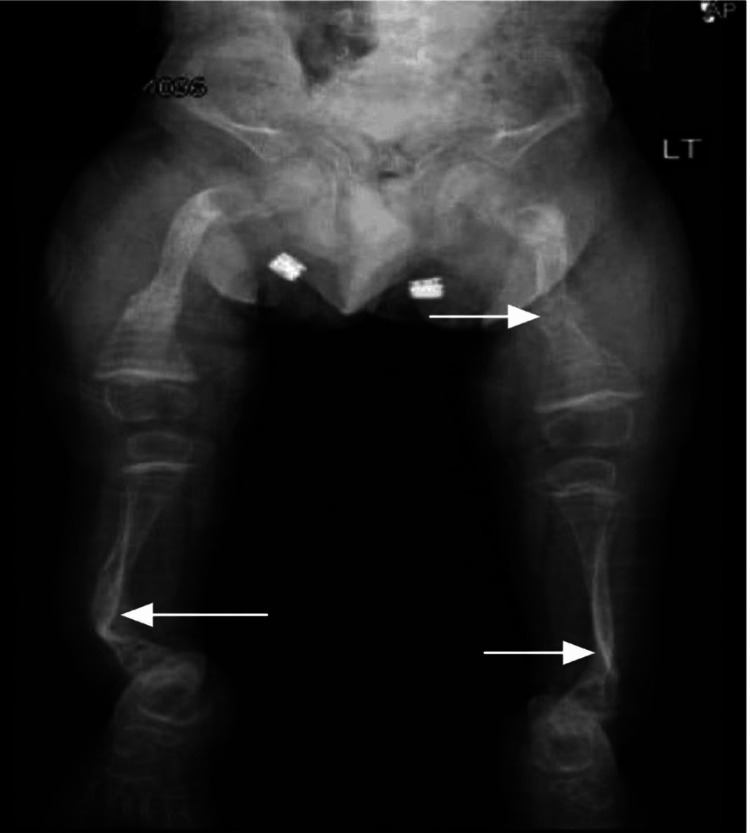
Plain radiograph (anteroposterior view) showing short and wide bones, thin cortices, rarefaction of trabecular bone, and osteopenia. Various bone deformities, including anterior bowing of the tibia (saber shin deformity), are observed. Arrows indicate multiple fractures in different stages of healing.

A three-year-old male with OI presented with a spontaneous left femur fracture, necessitating ordering a radiograph (Figure 1). He appeared well, active, and positioned comfortably, with no signs of cyanosis or dehydration. Physical examination revealed blue sclerae, a triangular face, transparent, short, broken teeth, and shortened upper and lower limbs.

He was delivered via cesarean section due to a breech presentation to a 21-year-old primigravida, showing several skeletal abnormalities. He did not cry immediately upon birth but responded to oronasal suctioning and tactile stimulation. The mother denied any trauma or abuse during pregnancy. Both parents are second-degree cousins, with no significant family history. Routine antenatal ultrasounds revealed deformed lower limbs.

At birth, he experienced moderate respiratory distress, prompting the immediate initiation of newborn resuscitation measures, including non-invasive respiratory support, such as nasal cannula and mask ventilation. Recognizing the need for advanced monitoring and intervention, he was transferred to the Neonatal Intensive Care Unit (NICU). The treatment plan comprised mask ventilation, antimicrobial therapy, infusion therapy, parenteral nutrition, and adequate analgesia. He showed steady improvement and transitioned to spontaneous breathing within three days. Cranial examination revealed macrocephaly with a characteristic triangular facial configuration. The sclerae showed a bluish color, with no signs of pallor or jaundice. The anterior fontanelle was in its normal position with open sutures, and muscle tone remained within normal limits. Significant skeletal abnormalities were noticeable in both upper and lower extremities: bowing of femurs and tibiae with metaphyseal flaring, along with deformities in the ulna and radius; multiple fractures were evident in the tibiae, radii, and femurs; his chest appeared constricted with shortened ribs; and a genital examination revealed bilateral asymmetrical scrotal swelling. Anthropometric measurements indicated a normal birth weight of 3150 grams, a length of 43 cm, placing him at the first percentile, and a head circumference of 38 cm, falling at the 99th percentile. After monitoring and confirming clinical stability, he was discharged successfully at nine days old.

Clinical and radiographic assessments established the primary diagnosis of OI type III for the child, subsequently confirmed by whole-exome sequencing (WES) that revealed the patient's heterozygosity for a likely pathogenic variant in the COL1A2 gene: p.6322V. This variant, previously unreported as either pathogenic or benign, occurs within the conserved Gly-X-Y triple helical region across species. It is absent from population databases.

A missense variant at the same residue (c.964G>A (p.Gly3225er)) has an association with OI (ClinVar entry: 61802). In silico analysis suggests probable damage to the protein's structure and function. Hence, this variant is likely pathogenic. He also carries a homozygous variant of unknown clinical significance in the LAMB1 gene. This variant is documented in population databases (rs193010498, gnomAD 0.2%).

Family members underwent WES targeting the COL1A2 variant to assess familial recurrence risk, revealing that both parents lacked the mutated allele in the COL1A2 gene. The treatment regimen involved pamidronate administration, starting at seven months with a dose of 3 mg over six hours infused with 100 cc of normal saline for three days every three months, with monitoring calcium levels before and after administration (Table 1).

**Table 1:** Calcium levels before and after administration of pamidronate for the last six doses of the drugs.

Dose	Calcium level before administration in milligrams/deciliter (mg/dl)	Calcium level after administration in milligrams/deciliter (mg/dl)
First	10.3	10.2
Second	9.9	9.8
Third	9.7	9.6
Fourth	9.8	9.7
Fifth	9.3	9.4
Sixth	9.5	9.6

## Discussion

OI, known as brittle bone disease, is a hereditary connective tissue disorder with genetic and clinical implications. It primarily stems from a genetic impairment affecting bone quality, leading to recurrent fractures, short stature, blue sclera, triangular face, barrel rib cage, curved spine, vertebral collapse, brittle teeth, hearing loss, and respiratory issues [6,7]. Consequently, our patient underwent hearing and ophthalmic examinations at the age of three years.

Sillence introduced a classification system categorizing OI into at least 19 types, ranging from type I to type XIX, based on clinical and radiological features and inheritance mode [8,9]. Type I is the mildest form, affecting approximately 60% of cases with bone fragility and a normal lifespan. Type II, the most severe, seen in about 10% of cases, is often fatal shortly after birth due to multiple fractures. Type III, accounting for approximately 20% of cases, is severe, with significant disability and a shortened lifespan. Type IV, in around 6% of cases, is moderate, requiring walking aids but with a near-normal lifespan and normal sclerae. Types V to XIX are rarer and encompass a spectrum of manifestations from moderate to severe bone fragility, growth deficiencies, skeletal deformities, and other specific clinical features, each representing a small percentage of OI cases. This classification system aids in understanding the diverse clinical presentations of OI, guiding diagnosis and management strategies accordingly [7,10].

OI is mostly caused by mutations in genes for collagen protein (COL1A1 and COL1A2). These mutations frequently replace glycine with another amino acid, disrupting collagen structure and influencing the severity of OI. Other mutations, such as frameshifts, splice site alterations, and nonsense mutations, can also play a role in OI development [11].

Cardiopulmonary insufficiency and respiratory infections are the primary reasons for morbidity and mortality associated with OI after birth. These issues arise from changes in lung collagen and complications related to thoracic skeletal dysplasia, including scoliosis and rib cage deformities limiting pulmonary function [4]. In our case, an echocardiogram done at birth showed a small atrial septal defect in the secundum, and a repeat at age 10 months showed no anatomical problem, normal function, and moderate pulmonary hypertension (systolic 60 mmHg), prompting treatment initiation with Lasix 2 mg/kg/day and aldactone 2 mg/kg/day.

Prenatal ultrasound can detect bone abnormalities and other signs of OI, like being small for gestational age. Further evaluation may be needed if long bone measurements are below the fifth percentile or polyhydramnios are present [4]. In this case, a detailed ultrasound was not done.

Fetal DNA testing for OI mutations is recommended if the condition runs in the family; amniocentesis or chorionic villus sampling (CVS) is used to get a sample of fetal cells [12], which was not done in our case due to financial issues.

Studies show that cesarean sections for OI do not reduce fractures, but factors like breech presentation and maternal OI influence delivery choice [4]. In this clinical case, the CS was chosen because of the breech fetal presentation.

While OI lacks a cure, treatment focuses on preventing fractures and deformities. The management approach typically includes medications, physical therapy, and surgical intervention when necessary [7].

Physical therapy plays a key role in strengthening muscles, improving flexibility, and teaching safe movement strategies to minimize fracture risk. Bracing the limbs and spine can also provide support and potentially prevent deformities. In severe cases, surgery may be required to address bone malformations. These procedures can involve inserting rods for stabilization, repositioning bones through osteotomies, or fusing vertebrae in the spine. The specific treatment plan is tailored to each patient's unique needs and condition [7].

In our patient, treatment with pamidronate begins at seven months of age. Vitamin D and calcium supplements are also well-tolerated and do not cause any complications. Finally, there is a limitation in our patient's follow-up because he comes to the hospital every six months for pamidronate injections.

## Conclusions

Early identification and intervention are essential for effective management of OI. Timely prenatal ultrasound screening, followed by confirmatory genetic testing, such as exome sequencing, allows for prompt initiation of appropriate interventions, like pamidronate therapy.

This multifaceted approach, involving close collaboration across various medical disciplines, is pivotal in enhancing patient care and outcomes for those living with this complex genetic disorder.
